# p53 elevation in human cells halt SV40 infection by inhibiting T-ag expression

**DOI:** 10.18632/oncotarget.10769

**Published:** 2016-07-21

**Authors:** Nir Drayman, Orly Ben-nun-Shaul, Veronika Butin-Israeli, Rohit Srivastava, Ariel M. Rubinstein, Caroline S. Mock, Ela Elyada, Yinon Ben-Neriah, Galit Lahav, Ariella Oppenheim

**Affiliations:** ^1^ Department of Hematology, Hebrew University Faculty of Medicine and Hadassah University Hospital, Jerusalem, Israel; ^2^ Department of Systems Biology, Harvard Medical School, Boston, Massachusetts, USA; ^3^ The Lautenberg Center for Immunology and Cancer Research, Hebrew University Faculty of Medicine, Jerusalem, Israel; ^4^ Department of Cell Biology, Weizmann Institute of Science, Rehovot, Israel

**Keywords:** p53, SV40, large T-antigen, Sp1, host defense

## Abstract

SV40 large T-antigen (T-ag) has been known for decades to inactivate the tumor suppressor p53 by sequestration and additional mechanisms. Our present study revealed that the struggle between p53 and T-ag begins very early in the infection cycle. We found that p53 is activated early after SV40 infection and defends the host against the infection. Using live cell imaging and single cell analyses we found that p53 dynamics are variable among individual cells, with only a subset of cells activating p53 immediately after SV40 infection. This cell-to-cell variabilty had clear consequences on the outcome of the infection. None of the cells with elevated p53 at the beginning of the infection proceeded to express T-ag, suggesting a p53-dependent decision between abortive and productive infection. In addition, we show that artificial elevation of p53 levels prior to the infection reduces infection efficiency, supporting a role for p53 in defending against SV40. We further found that the p53-mediated host defense mechanism against SV40 is not facilitated by apoptosis nor via interferon-stimulated genes. Instead p53 binds to the viral DNA at the T-ag promoter region, prevents its transcriptional activation by Sp1, and halts the progress of the infection. These findings shed new light on the long studied struggle between SV40 T-ag and p53, as developed during virus-host coevolution. Our studies indicate that the fate of SV40 infection is determined as soon as the viral DNA enters the nucleus, before the onset of viral gene expression.

## INTRODUCTION

p53 was originally discovered as a host protein associated with SV40 large T-antigen (T-ag) [1, 2]. It was later found to be a major tumor suppressor, mutated in ~50% of isolated tumor tissues [3, 4]. p53 has been described as “guardian of the genome” due to its central role in keeping genome integrity in response to genotoxic stress [5, 6] as well as many other insults [7]. As a transcription factor [8] p53 activates or represses a variety of target genes, leading to DNA repair, cell-cycle arrest, senescence, or apoptosis [9, 10]. The choice of p53 target genes depends on p53 protein levels [11], modification state [12, 13] [14, 15], and its temporal behavior [16] [17].

Being a central cellular sensor of external insults, the activity of p53 is regulated by multiple posttranslational modifications, including phosphorylation, acetylation and ubiquitination affecting its stability and activity [18]. A major regulator of p53 is the E3 ubiquitin ligase Mdm2, which leads the protein to proteosomal degradation [19, 20]. p53 induces Mdm2 transcription, creating a negative feedback loop that keeps p53 at a low steady state level in the population [21, 22] with sporadic, spontaneous pulses of p53 in single cells [23], and a series of pulses in response to DNA damage [24, 25].

SV40 is a small, non-enveloped DNA virus belonging to polyomaviridea. Its genome is a 5.2 KB circular double stranded DNA. The viral T-ag is a major regulator of the viral life cycle, as its expression is absolutely required for initiation of viral DNA replication, propagation of viral progeny and cell lysis. T-ag is expressed soon after nuclear entry of the viral genome, during the early phase of the infection. It regulates its own transcription by feedback inhibition [26] as well as the transition from early to late gene expression by activating the late promoter [27]. The late phase of infection includes biosynthesis of the capsid proteins, virion assembly and induction of necrotic cell lysis [28, 29].

SV40 is readily propagated in the laboratory, and has therefore served as a paradigm for studying polyomaviruses, including the known human pathogens BK and JC [30]. Polyomaviruses are abundant in birds and mammals, with a highly conserved structural organization. Most are restricted to specific hosts and cells, due to limited receptor recognition and different requirements for cellular regulatory factors. In recent years new polyomoviruses have been isolated from human tissues, bringing the number of human members of the family to ten [31]. At least two of these appear to be associated with human diseases, Merkel cell polyomavirus (MCV) [32] and trichodysplasia spinulosa-associated polyomavirus (TSV) [33].

SV40 infection is a slow process. The virus enters the cells via endocytosis, by binding to ganglioside GM1 in caveolar/lipid raft domains at the plasma membrane [34-36]. SV40 also adsorbs to a number of cellular protein receptors [37] accounting for its wide tropism and for the induction of a complex signaling network [38, 39]. The virus traffics via the endosomal pathway into the ER [40], where it disassembles at 5-6 hours [41, 42]. The free minichromosome enters the nucleus by direct passage from the ER through viroporins in the inner nuclear envelope [43-45]. Nuclear entry is at ~8 hours [44]. Protein phosphorylation arrays revealed that during the first 6 hours of SV40 infection the virus elicits concurrently two opposing pathways: pro-apoptotic, via PARP-1 and p53, and anti-apoptotic, via Akt-1 and Hsp70 [39]. Remarkably the pathways are robustly balanced, as the infected cells neither apoptose nor proliferate. Akt-1 is absolutely required for the infection and its inhibition completely eliminated expression of T-ag [39].

Here we demonstrate that unlike Akt-1, p53 participates in host defense. Induction of apoptosis is a common host defense mechanism elicited by p53, viewed as an “altruistic” measure taken by infected cells to save the organism. However in the case of SV40, p53 activation does not lead to apoptosis. Instead we revealed that p53 protects cells from SV40 infection by a new mechanism; it binds to the viral DNA when it enters the nucleus and interferes with the progress of infection by repressing T-ag transcription.

## RESULTS

### p53 is activated following SV40 infection

**Figure 1:**
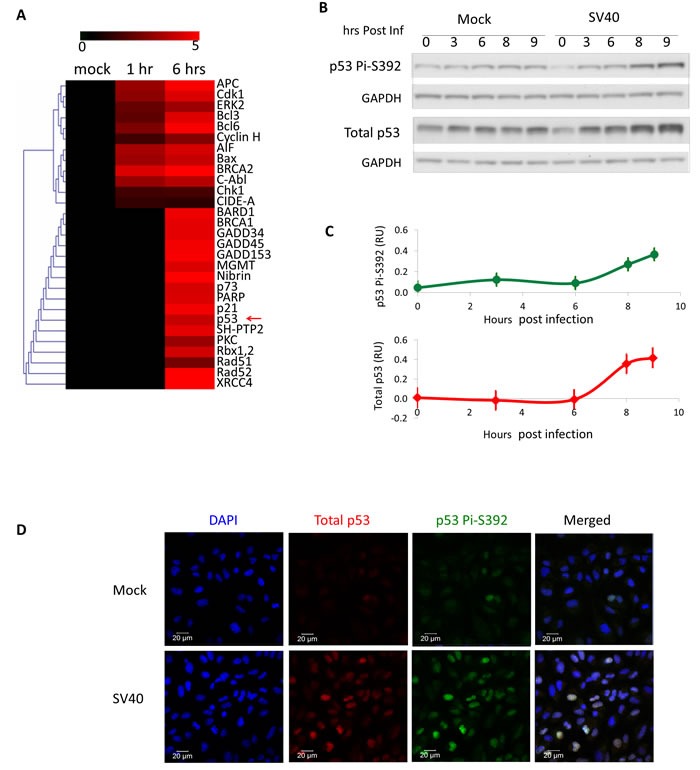
SV40 infection triggers activation of p53 **A.** Serine-phosphorylation of 29 proteins annotated to participate in DNA-damage signaling was probed in mock-infected and SV40-infected (moi 10) CV-1 cells using antibody arrays [39]. Red colors indicate increased phosphorylation compared to mock-infected cells. p53 is marked with a red arrow. **B.** Western blot analyses of whole cell lysates detecting total p53 and p53 phosphorylation at S392. **C.** Quantification of 3 independent infection experiments. The bands were quantified and the levels of total p53 and S392 bands were normalized to GAPDH. Since the level of total p53 increased also in the mock, presumably due to their approaching contact inhibition, we subtracted the values of the normalized bands of the 3 mock infections from their corresponding bands of SV40 infection. The same was done for S392 bands. The graph depicts average of 3 experiments; standard errors are represented by bars. **D.** Immuno-histochemistry of CV-1 cells. Cells were co-stained for total p53 and for p53 phosphorylated at S392, 9 hours post infection by SV40.

We have previously reported the use of antibody arrays to monitor serine phosphorylation events following SV40 infection of CV-1 cells [39]. Of the total 370 proteins identified in that screen, 29, including p53, participate in the DNA damage response (Figure 1A). p53 is phosphorylated at a moderate level at 6 hours post infection. To validate and further explore this finding we infected CV-1 cells and analyzed whole cells lysates at several time points. Western blotting (Figure 1B) indicated that at 8 and 9 hours post infection level of total p53 rises significantly above the mock (*p* = 0.05 and 0.04 respectively) and it becomes phosphorylated at S392 (*p* = 0.02 and 0.002, respectively). p53 phosphorylation parallels an increase in the p53 protein, suggesting that SV40 infection leads to p53 induction as well as activation. S392 phosphorylation is associated with enhancement of p53 binding to DNA [46-48] and tetramer formation [49]. We have not seen phosphorylation at S15 (Figure S1), which functions in p53 transcriptional activation [50].

Activation of the transcription factor p53 is associated with its nuclear localization. Immunostaining of CV-1 cells 9 hours post infection (Figure 1D) demonstrates that in some of the cells p53 level is increased and the protein is localized to the nucleus, consistent with its activation. Furthermore, the same cells also stain positive for phosphorylated S392, implying activation by S392 phosphorylation. The representative images demonstrate wide-ranging cellular heterogeneity with respect to p53 staining. However, screening many fields we observed that elevated p53 was always localized to the nucleus and consistently merged with S-392 phosphorylation.

### The role of p53 in SV40 infection

Our previous studies demonstrated that SV40 activates proteins that are required for its infection, such as PLC-gamma, Akt-1 and caspases 6 and 10. Inhibition of any of those led to elimination of T-ag expression [39]. Our working hypothesis was that in analogy to those proteins, p53 would also be required for the infection to proceed. Since a specific efficient inhibitor for p53 is not available [51, 52], we instead increased p53 level by treating cells with the Mdm2 inhibitor Nutlin3 [53].

**Figure 2:**
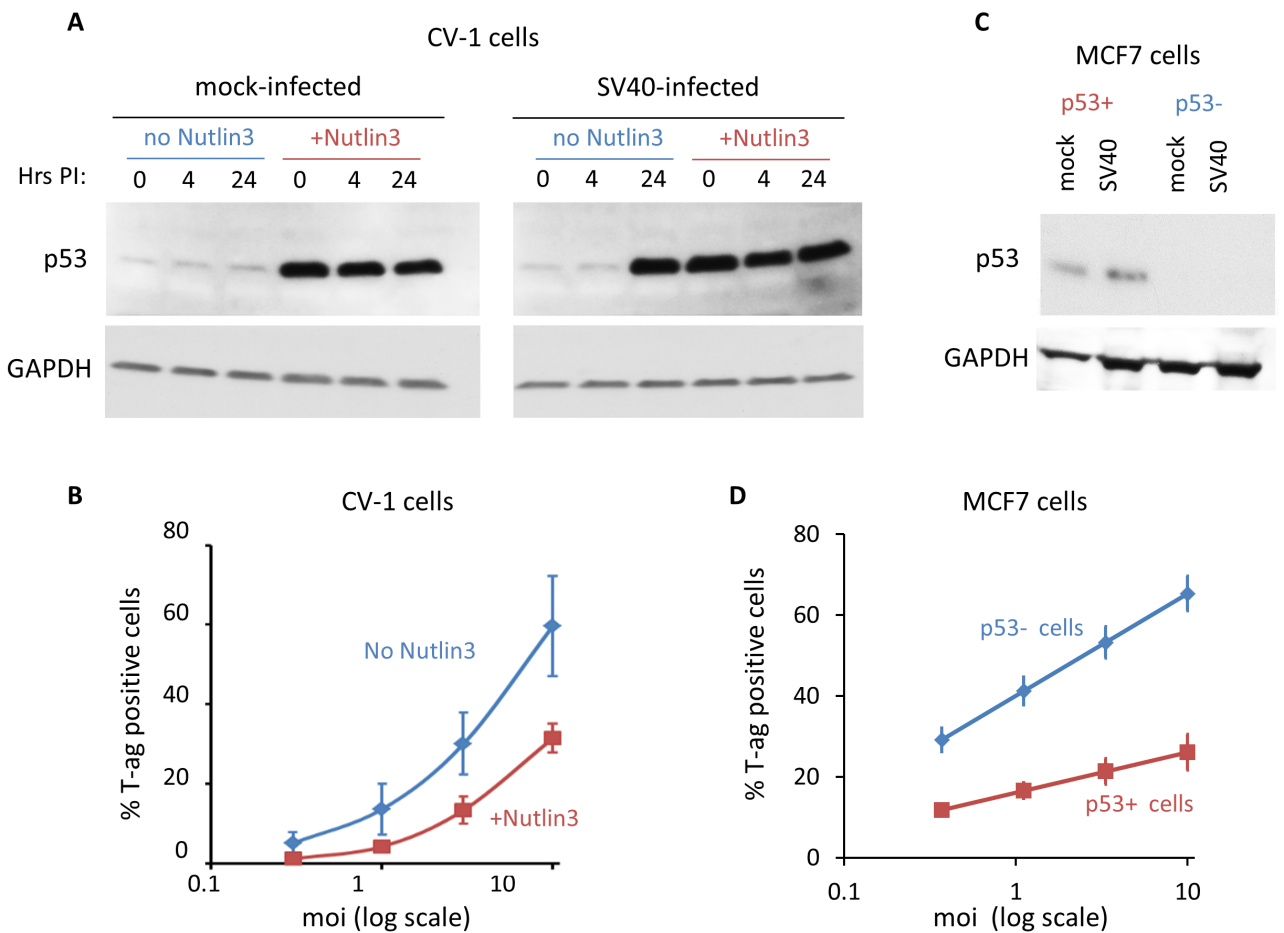
p53 functions in host defense against SV40 **A.** CV-1 cells were pre-treated with 20 μM Nutlin3 in 1% DMSO, or with 1% DMSO without Nutlin3, for 16 hours before infection. The western blot shows that Nutlin3 treatment dramatically increased p53 levels. At 24 hours post infection p53 is elevated in infected cells due to the infection and regardless of Nutlin3 treatment. **B.** SV40 infectivity following Nutlin3 pre-treatment in CV-1 cells. Cells treated as in panel A were infected at several moi's and the percentage of infected cells was determined 24 hours post adsorption by FACS staining of T-ag, as previously described [56]. Data points represent mean ± S.E. of three independent experiments. The decrease in infection of the Nutlin3 treated cells is statistically significant (*p*-value < 0.01, one-sample t-test). **C.** Western blot showing p53 level in p53-positive and negative MCF7 cells, harvested 24 hours post infection. As expected, there is no p53 in the negative cell-line. **D.** SV40 infectivity of the MCF7 cell lines. Cells were infected at several moi's and the percentage of T-ag positive cells was determined 24 hours post adsorption. The increase in infection of the p53 negative cell line is statistically significant (data points for 3 independent experiments; *p*-value < 0.01, one-sample t-test).

Western blotting experiments indicated that p53 levels were significantly increased (by approximately 50-fold) following 16 hours treatment with 20 μM Nutlin3 of both mock and SV40-infected CV-1 cells (Figure 2A). Therefore in the following experiments cells were pre-treated with Nutlin3 for 16 hours prior to the infection, and Nutlin3 was re-added to the medium following the adsorption period. Note that SV40 infection, regardless of Nutlin3 treatment, results in accumulation of p53 late in infection (at 24 hours), as was previously reported [54, 55].

Assuming that p53 is needed for the infection, we expected that Nutlin3 pre-treatment would increase SV40 infection rate, measured as the percentage of T-ag positive cells at 24 hours post infection by FACS. As the number of T-antigen positive cells at 48 hours accurately represents the viral titer [56], we used this method as a measure for infection level. To our surprise Nutlin3 significantly reduced the percentage of T-ag positive cells (Figure 2B), suggesting that p53 hinders the infection rather than supporting it. At moi 0.3 Nutlin3 reduced SV40 infectivity from 5 to 1.2% T-ag positive cells and at moi 10 it was reduced from 60 to 31%. These results suggested to us that early in the response to SV40 infection p53 participates in host defense.

We further validated the potential host protective role of p53 by reducing its level in the human breast-cancer MCF7 cells. We used a pair of MCF7 cell-lines, p53 positive and p53 negative, the latter derived by stable transfection with shRNA against p53 [57]. We found that the proliferation rate of both cell-lines was similar, with a generation time of ~24 hours, until they reach ~70% confluence (Figure S2). Beyond that point proliferation of the p53-positive cells began to slow down significantly, while the p53-negative cells continued to proliferate at the same rate as before.

Cells carrying sh-p53 had no detectable levels of p53 either in mock or SV40-infected cells (Figure 2C). The absence of p53 in these cells led to a ~50% increase in their susceptibility to SV40 infection at moi ranging from 0.3 to 10 (Figure 2D). These results support an anti-SV40 activity of p53 as was suggested by the results in CV-1 cells.

### p53 activation and its effect on SV40 infection in single cells

Our immunostaining experiments revealed that p53 activation is not uniform throughout the culture. In some infected cells nuclear p53 is abundant while in others it is hardly visible (Figure 1D). The multiplicity of infection (moi) in these experiments was 10 plaque-forming units per cell (pfu/cell). SV40 infectivity is low, and each pfu represents ~200 DNA-containing virions [39, 58]. At this multiplicity of infection, over 90% of the cells internalize the virus (Table S1). Furthermore, treatment with Nutlin3 had only a minimial effect on viral entry (83% positive cells compared to 94% in the untreated cells, Table S1), which by itself cannot explain the pronounced effect of Nutlin3 treatment on T-ag expression (23% positive cells compared to 78% in the untreated cells, Table S1). Thus the considerable cellular heterogeneity in p53 is unlikely to be related to variability in cellular entry of the virus.

This wide heterogeneity in p53 levels six hours after infection could be interpreted in at least two ways: i. only a subset of the infected cells activate p53 during the infection; ii. activation of p53 is transient and not synchronized, occurring at different times in different cells. To better understand p53 dynamics at the individual cell level we performed time-lapse imaging of SV40-infection of an MCF7 cell-line that expresses a p53-venus fusion protein. The p53-venus of this clone was previously shown to faithfully mimic the behavior of endogenous p53 as determined by western blots of cells in response to different stress signals [16, 23, 25].

**Figure 3:**
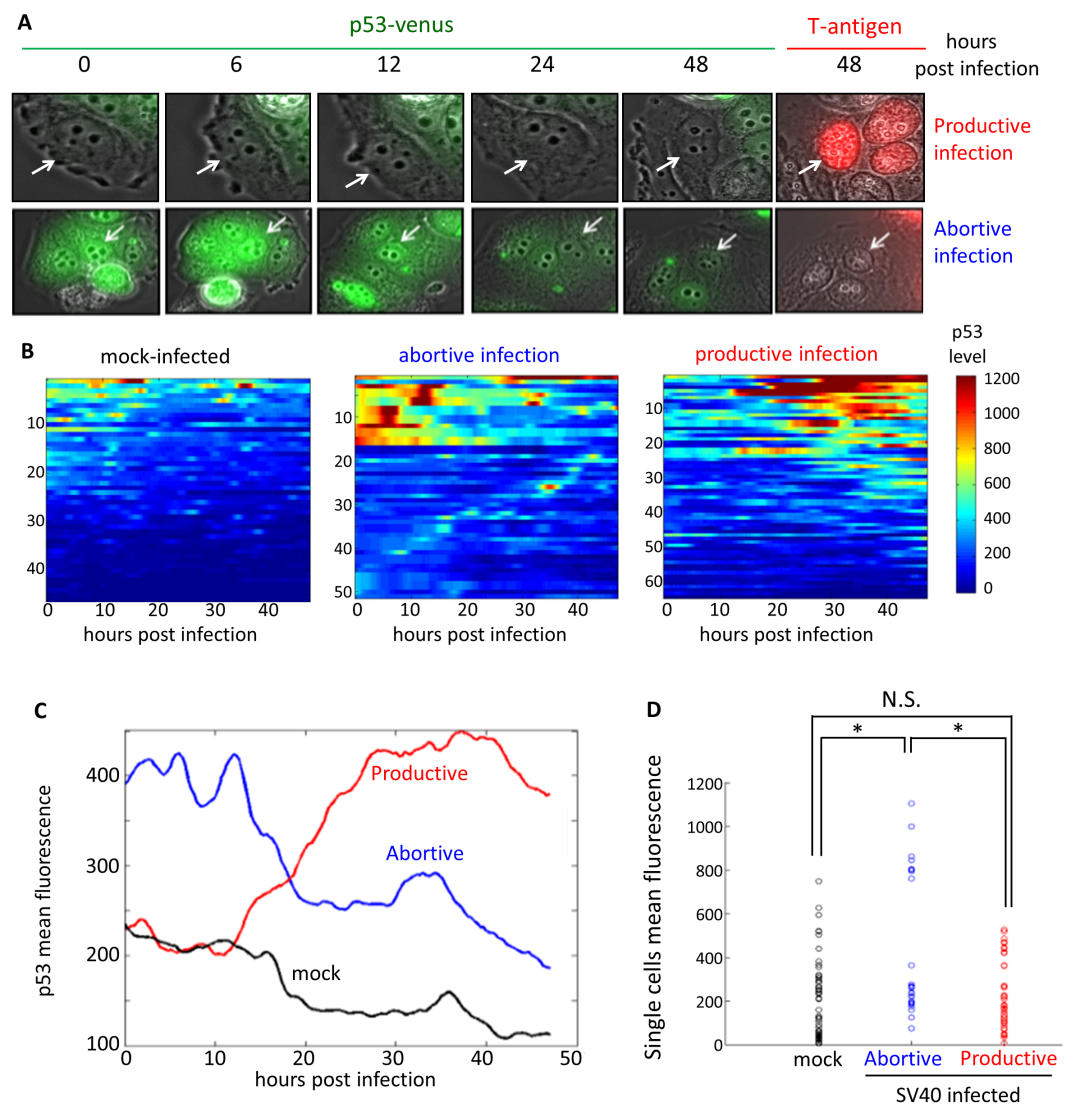
Dynamics of p53 in single cells following SV40 infection MCF7 cells carrying a p53-venus fusion protein were infected at moi 30. Images were taken every 20 min for 48 hours, at which time the cells were fixed and stained for T-ag. The data represent tracked cells from 4 independent experiments **A.** Representative images of SV40-infected cells. p53-venus appears in green and T-ag staining in red. The upper panels show a productively infected cell (white arrow, T-ag positive at 48 hrs) at the indicated time points; the lower panels show abortive infection (white arrow). **B.** Heat-maps representing the dynamics of p53 fluorescence in individual cells. Data include 46 mock infected (left panel) and 117 SV40-infected, including 51 abortively infected (middle panel) and 66 productively infected MCF7 cells (right panel). Each row represents a single cell. Relative p53 level in each cell over time is evaluated by the level of Venus fluorescence. The data are color-coded, from blue (low levels) to red (high levels). Analyses of the data are presented in panels (C) and (D). **C.** Mean p53 level of each group of cells, in arbitrary units determined as mean fluorescence, is represented over time. See S3 Figure for zoom in on the data for the first 12 hours after infection. **D.** Mean fluorescence of individual cells, represented by small circles, during the first 12 hours of infection. p53 fluorescence in the mock and productively infected cells are similar (*p*-value = 0.99, two-tailed t-test). Abortively infected cells are clustered into two subgroups, with high and low p53 levels. p53 fluorescence of the abortively infected cells (both subgroups taken together) is significantly different from both mock and productively infected cells (*p*-value < 0.001, one-tailed t-test).

Since MCF7 cells are about 3-fold less susceptible to SV40 infection than CV-1 [59] infections in subsequent experiments were performed at moi 30. (equivalent to ~6000 virion particles/cell), to ensure that the virus entered most of the cells. The infected cells were cultured for 48 hours in a microscope chamber under controlled conditions, and images were acquired every 20 minutes. At the end of the experiment the cells were fixed and stained for T-ag. The infected cells were therefore classified into two categories - productively infected (T-ag positive at 48 hours) and abortively infected (T-ag negative). Figure 3A shows snapshots of cells at different times after infection of a productively infected cell (upper panel) and an abortively infected cell (lower panel). Mock-infected cells were similarly treated and analyzed.

Analysis of 46 mock-infected cells confirmed no major changes in p53 levels in the majority of those cells (Figure 3B, left panel). A small number of the mock-infected cells showed short pulses of p53, which resembled the previously described spontaneous p53 pulses in actively dividing cells [23]. The cellular heterogeneity with respect to p53 induction was reproduced in this experiment as well. We followed p53 levels in a total of 117 SV40 infected cells. T-ag staining revealed that 66 cells (~56%) were productively infected, while in the other 51 cells (~44%) the infection was abortive. Strikingly, all the cells with high levels of p53 during the first 12 hours of infection aborted the infection (Figure 3B, middle panel), while none of the cells with productive infection had high p53 during that period (right panel). This finding underscores the host-protective role of p53 against SV40 during the first phase of the infection. Note the presence of abortively infected cells with low p53 levels during the first 12 hours. These cells suggest the existence of other, p53-independent anti-viral factors that block SV40 infection. Alternatively, it is possible that in these cells the infection was aborted due to elevation of endogenous p53, which was not measured in these experiments.

As seen in Figure 3B, some of the productively infected cells showed significant levels of p53 later in the infection, when T-ag was already present, consistent with early reports demonstrating that p53 accumulates in SV40 infected cells [54, 55], but is rendered inactive by T-ag [60].

Figure 3C depicts the mean fluorescence of p53 for each group for the entire 48 hours of the experiment. For clarity the data for the first 12 hours, which appear to be crucial for the decision between infection progress versus its arrest, are expanded in Figure S3. During that time period abortively infected cells show a higher mean fluorescence than either productively infected or mock-infected cells. This analysis accentuates the switch that occurs at 12-20 hours, when the average p53 level in the productively infected cells increases while it decreases in the abortively infected cells.

In another analysis the mean level of p53 for each individual cell during the first 12 hours was computed and is shown in Figure 3D. In agreement with Figure 3C and Figure S3, all the productively-infected cells exhibited low p53 levels, < 550 RU (relative units), which is similar to most of the mock-infected cells. The abortively infected cells on the other hand were grouped into two distinct subclasses, one with high p53 levels, 750-1150 RU, and the other with low p53, < 400 RU. p53 fluorescence of the abortively infected cells (both subgroups taken together) is significantly different from both mock and productively infected cells. This analysis highlights the role of p53, and possibly other unknown factors, in host protection. We have concluded that a bi-stable decision of the infection progress depends on p53 levels during the first 12 hours post infection. Furthermore, since the difference in p53 levels between productive and abortive infections already observed at time 0, we suggest that the progress of infection depends on the basal levels of p53 prior to the infection itself.

### What is the mechanism of p53 mediated cell defense?

p53 may lead to host protection by directing the infected cell to apoptosis, as shown for vesicular stomatitis virus (VSV), influenza A, herpes simplex (HSV) and poliovirus [61]. Another potential p53 defense mechanism, detrimental to viruses requiring cell division for infection, is cell cycle arrest.

**Figure 4:**
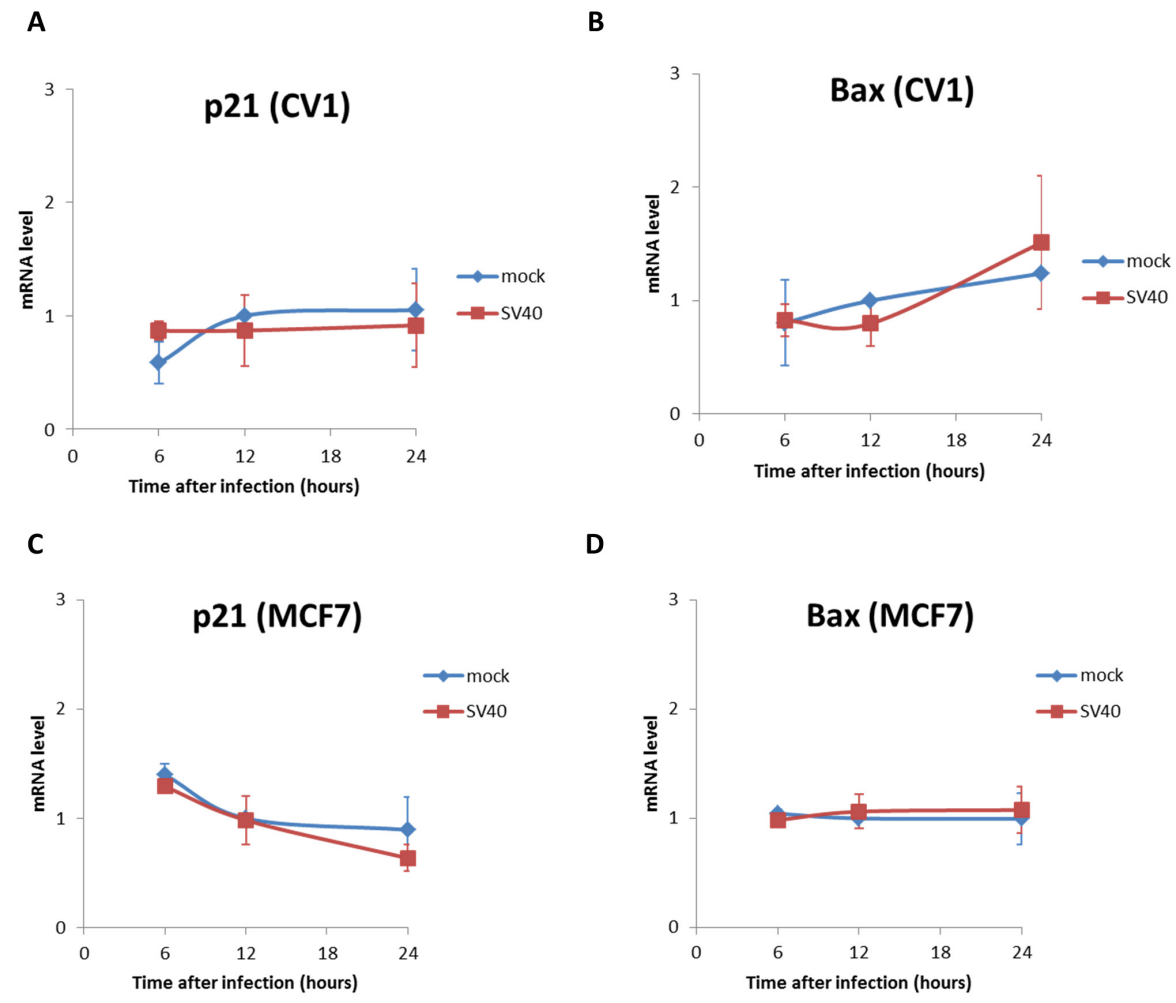
mRNA levels of the p53 target genes, p21 and Bax Mock and SV40-infected cells were harvest at 6, 12 and 24 hrs post infection and RNA was extracted. mRNA levels of the p53-target genes that regulate cell cycle and apoptosis were analyzed by quantitative RT-PCR. The primers are listed in S1 Table. **A.**-**B.** CV-1 cells; **C.**-**D.** MCF7 cells. The figure presents averages and standard error of 3 independent experiments. Levels of both Bax and p21 were not significantly different between mock and SV40-infected cells in both cell lines (p-value > 0.05, two-tailed t-test).

To test for these possibilities we asked whether the canonical p53 target genes Bax, a central regulatory factor of the apoptotic pathway, and p21, a cell-cycle arrest gene, are increased following SV40 infection. As seen in Figure 4, the infection does not lead to a significant rise of mRNA levels of either Bax or p21 with respect to the mock in both CV1 and MCF7 cells. These results suggest that neither apoptosis nor cell cycle arrest account for p53 host defense against SV40.

**Figure 5:**
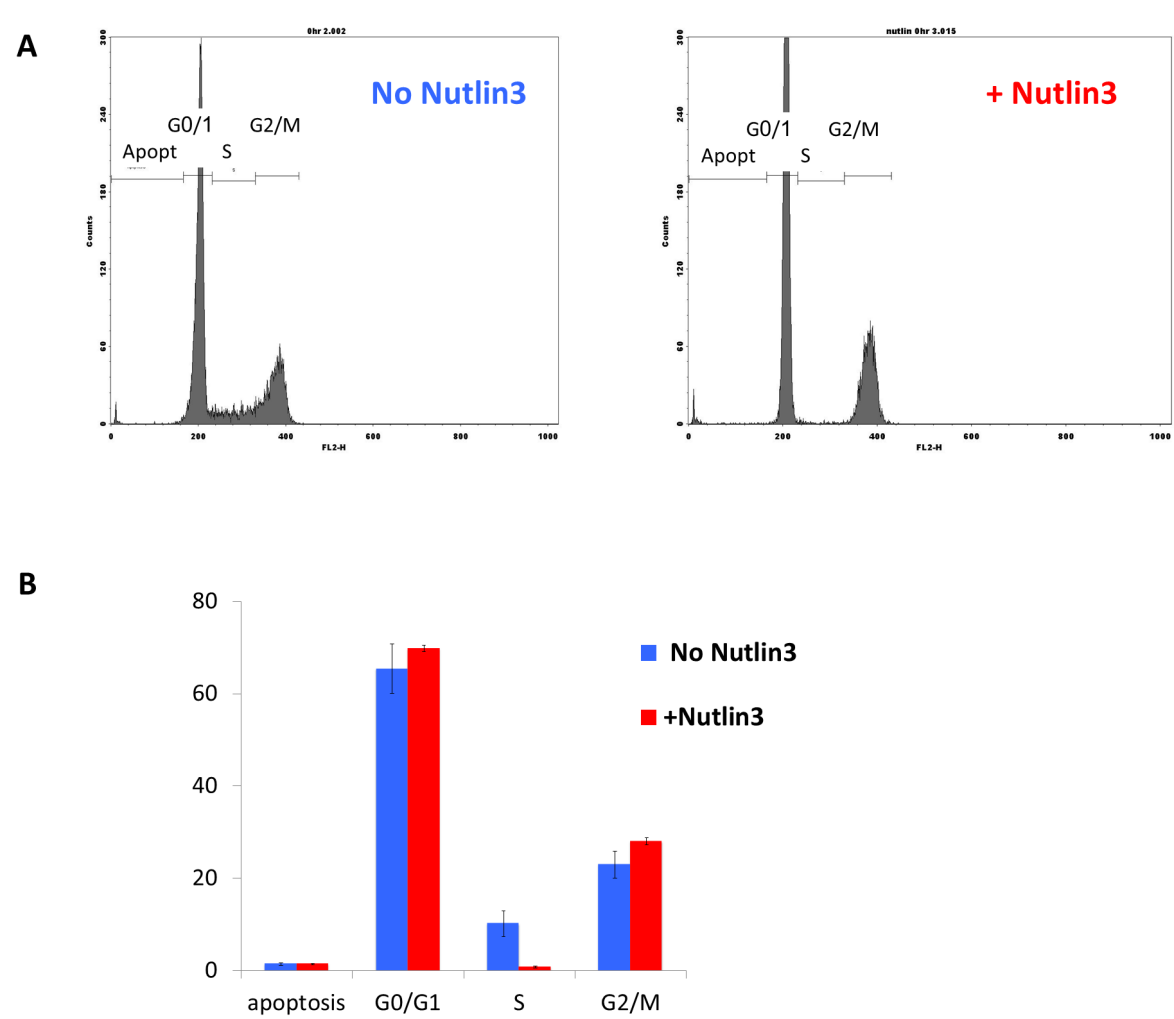
The effect of p53 elevation on the cell-cycle of CV-1 CV-1 cells were treated for 16 hours with 20 μM Nutlin3 in 1% DMSO (colored red in Figure 5B) or with 1% DMSO without Nutlin3 (colored blue in Figure 5B). The cells were harvested by trypsinization and fixed with 70% ethanol overnight. For FACS analysis the cells were treated with RNAse and stained with Propidium Iodide.

However, it was still feasible that p53 induces apoptosis or cell-cycle arrest via the activation of other cellular targets. We therefore asked whether the increase in p53 following Nutlin3 treatment affects the cell cycle or induces apoptosis in CV-1 cells. As seen in Figure 2A, 2B 16 hours pre-treatment with Nutlin3 elevates p53 level ~50 fold, leading to a reduction in SV40 infection at moi's of 0.3-10 by 50-75%. We asked whether Nutlin3 pretreatment affected CV-1 cell cycle at the time of infection. As seen in Figure 5, treatment with Nutlin3 for 16 hours did not significantly change the proportion of cells in G0/G1 (from 65 to 70%, *p*-values = 0.5), G2/M (from 23 to 28%, *p* = 0.2), or the percentage of apoptotic cells (1.43 to 1.40%, *p* = 0.9). The only significant Nutlin3 effect was a reduction in S-phase cells (from 10.2% without Nutlin3 to 0.8% with Nutlin3, *p* = 0.03). However, reduction in the proportion of S-phase cells at the time of infection is unlikely to account for the protective effect of p53, as SV40 does not rely on S-phase for entry or for its DNA replication [62].

Taken together, these results strongly suggest that the main effect of p53 elevation on the fate of the infection is not mediated by cell cycle modifications. Furthermore, the results are consistent with our previous studies, which demonstrated that SV40 infection does not induce apoptosis in CV-1 cells, analyzed at 9, 18 and 24 hours, way beyond the decision between abortive and productive infection (Figure 3 in [39]). In addition, apoptosis was not detected in the MCF7 cells imaged in the single-cell experiments for the 48 hours of the experiment. Lastly, SV40 readily infects quiescent cells and its nuclear entry does not depend on cell division [44, 45].

Another potential mechanism by which p53 may act as an anti-viral factor is through the induction of interferon-stimulated genes (ISGs) [63], as demonstrated for Sendai virus (SeV), Influenza and hepatitis C (HCV) [61]. In addition, ISGs were induced by ectopic expression of T-ag [64, 65] in mouse embryonic fibroblasts (MEFs), which are non-permissive for SV40 propagation. We proceeded to analyze whether ISGs are induced following infection by the wild type virus, of the SV40-permissive MCF7 and CV-1 cells.

**Figure 6:**
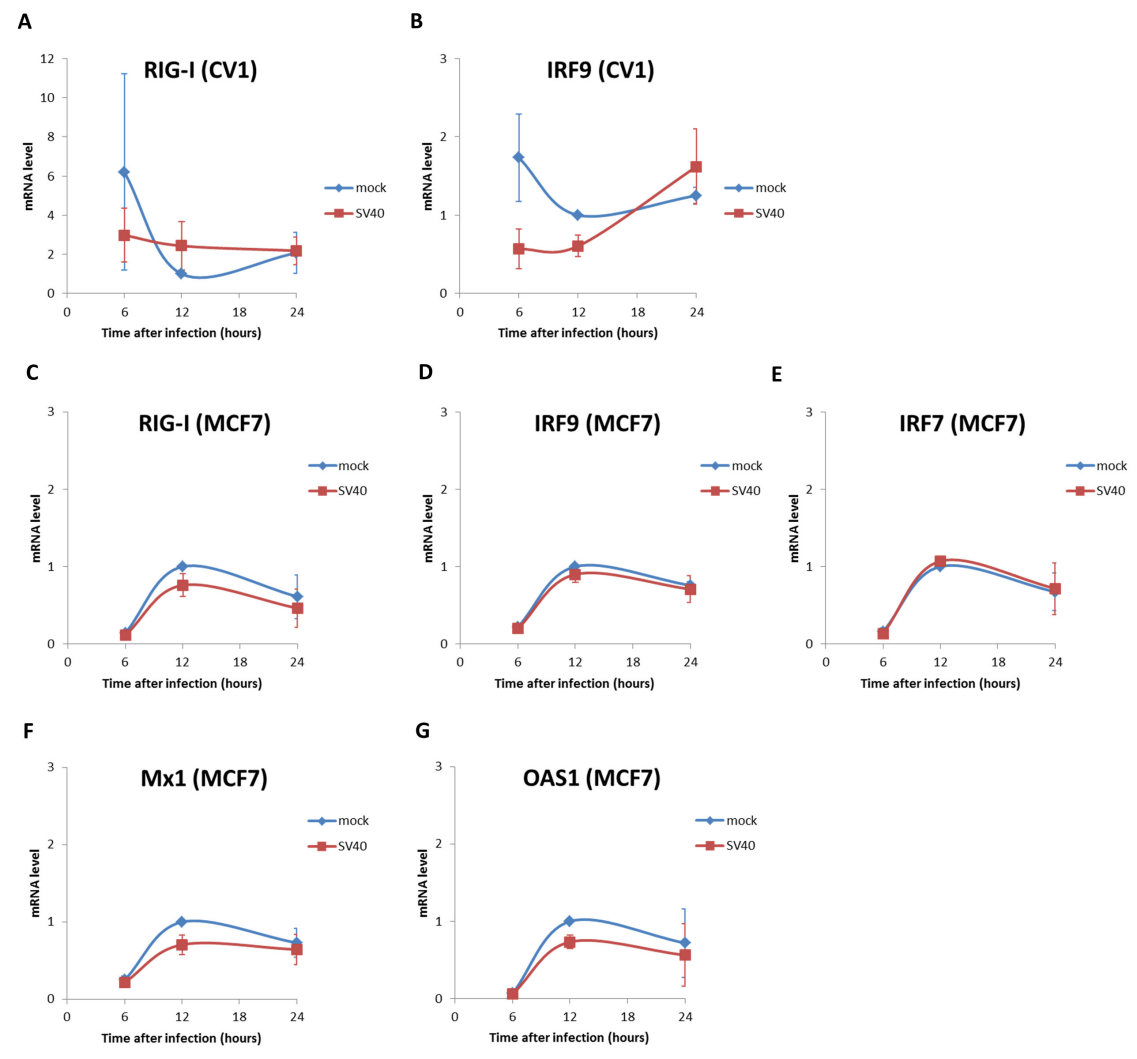
Interferon stimulated genes are not induced in SV40 infected cells Mock or SV40-infected cells were harvest at 6, 12 and 24 hrs post infection and RNA was extracted. mRNA levels of interferon stimulated genes were analyzed by quantitative RT-PCR. The primers are listed in S1 Table. **A.**, **B.** CV-1 cells; **C.**-**G.** MCF7 cells. The figure presents averages and error bars of 3 independent experiments. The mRNA levels of all the tested genes were not significantly different between mock and SV40-infected cells (*p*-value >0.05, two-tailed t-test). The only exception was IRF9 at 12 hours, when the mock mRNA was slightly higher (*p* = 0.044) than that of the infected cells.

We studied expression of 5 ISGs previously shown to be upregulated in a p53-dependant manner upon viral infections, Mx1, RIG-I, IRF7, IRF9 and OAS-1 [63, 66, 67]. Our experiments (Figure 6C-6G) show that mRNA levels of none of the five genes increased following infection of MCF7 cells above the level seen in the mock-infected cells. In CV-1 cells mRNAs of only two of the five genes could be amplified, either due to very low levels of expression or sequence difference between the human primers and the monkey genome. None of the mRNAs tested was higher in the SV40-infected cells compared to the mock control.

The disagreement between our data and The findings cited above, in which ISGs were induced following ectopic expression of T-ag in MEFs [64, 65], most likely results from the profound difference between viral infection and ectopic expression of a single gene. This explanation is in complete accord with our previous study [59], in which we found that SV40 evades immune attack by Natural Killer cells by down regulation of the stress-induced ligand ULBP1, while ectopic expression of T-antigen led to the opposite outcome, inducing the same stress ligand. Note that transcription of the late SV40 RNA is continuous, overlapping the early transcript. It most likely functions as antisense to T-ag mRNA in viral infection, but not in ectopic T-ag expression [68]. Hence ectopic expression of T-ag may lead to results that are non relevant (or even contradictory) to the context of infection with the wild type virus.

We concluded that neither apoptosis nor induction of ISGs is likely to operate in SV40-infected cells.

### p53 binds to the infecting SV40 DNA and down-regulates T-ag mRNA level

**Figure 7:**
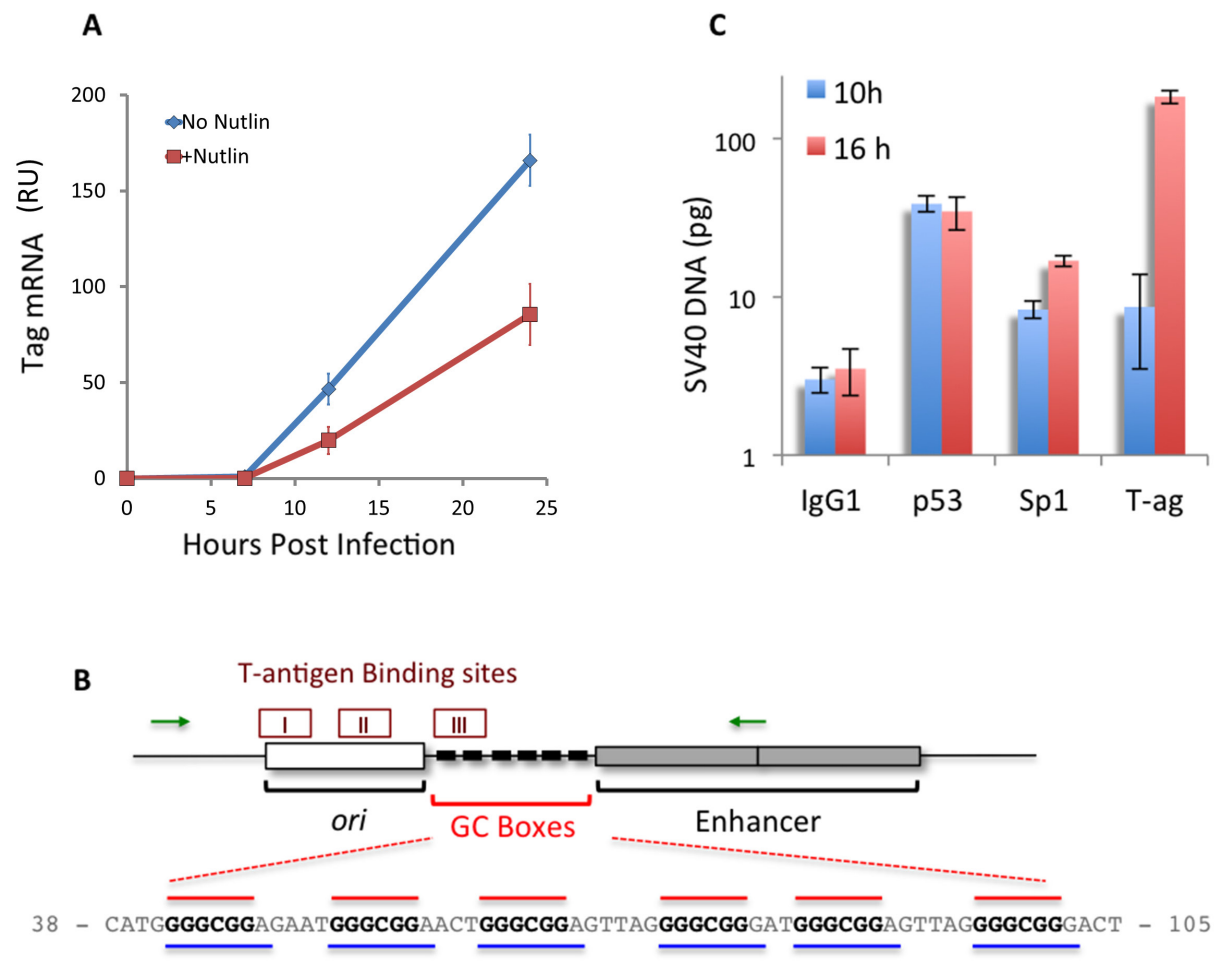
p53 binds to the SV40 early promoter, correlating with a decrease in T-ag mRNA **A.** CV-1 cells, with or without 16 hours Nutlin3 pre-treatment, were infected with SV40 and the level of T-ag mRNA, represented as relative units, was measured by quantitative RT-PCR at the indicated time-points, with HPRT RNA as an internal standard. Note that the T-ag protein is seen at 9 hours post infection (Figure S4). The results shown are mean ± S.E. of 5 independent experiments. For the statistical analysis, we compared the area under the curves and found that it was significantly lower in Nutlin3 treated cells compared to untreated cells (680±50 AU vs. 1400±142 AU, respectively. *p*-value = 0.004). **B.** Diagram of the regulatory region of the SV40 genome presenting the *ori* - origin of replication, the GC-boxes and the Enhancer, composed of duplicated 72 bp. The 3 T-ag binding sites are shown on top, and DNA sequence of the GC-boxes with the overlapping Sp1 (red) and p53 (Blue) binding sites below (http://alggen.lsi.upc.es/cgi-bin/promo_v3/promo/promoinit.cgi?dirDB=TF_8.3). The green arrows designate the location of the PCR primers used in the ChIP experiments. **C.** Binding of Sp1, p53 and T-ag to SV40 DNA *in vivo* was determined by ChIP at the indicated time points. DNA recovered from the immune precipitate was quantified by PCR with SV40 DNA as an internal standard. Results are mean ± S.E. of 3 independent experiments.

A number of viral and cellular promoters, including the early SV40 promoter and HIV promoter, were shown to be repressed by wild type p53 [69, 70]. We therefore asked whether the p53 host protection mechanism might be through direct repression of T-ag expression. This mechanism is consistent with the decrease in the percentage of T-ag expressing cells (Figures 2 and 3). We tested whether the p53 effect is at the level of T-ag transcription. To answer this question we quantified T-ag mRNA expressed under different levels of p53, manipulated via Nutlin3 treatment. As seen in Figure 7A, T-ag mRNA was not detected at 0 and 7 hours, before nuclear entry. Importantly, from 7 hours on, treatment with Nutlin3 significantly decreased the level of T-ag mRNA (*p*-values = 0.03 and 0.009 for 12 and 24 hours, respectively). Although we have not ruled out that Nutlin3 enhances T-ag mRNA degradation, this possibility seems unlikely. Regardless, expression of T-ag is dramatically reduced when p53 level is increased ~50 fold by Nutlin3 pre-treatment (Figure 2A).

T-antigen expression is activated via Sp1 binding at the GC-box region in the early promoter [71]. p53 was shown to bind at the same region *in vitro* [72] and *in vivo* [73], through sites that overlap with those of Sp1 binding (Figure 7B). Furthermore, *in vivo* binding of p53 reduced expression of a reporter gene (*cat*) by ~20 fold [69]. In addition, nuclear extracts containing p53 were shown, by gel-shift assays, to inhibit binding of Sp1 [73]. We hypothesized that p53-binding at the promoter underlies the decrease in T-ag expression.

To test the hypothesis we used chromatin immuno-precipitation (ChIP) and analyzed for *in vivo* binding of p53 and Sp1. We also analyzed for the binding of T-ag, since it binds to its own promoter functioning in negative autoregulation [26], and to the SV40 *ori*, for activating viral DNA replication [74], both required for progression of lytic infection.

In our hands T-ag begins appearing at a very low level (presumably in only few of the cells) at 9 hrs post infection (Figure S4), shortly after nuclear entry at 7-8 hours. The experiments were therefore performed at 10 and 16 hours post infection, during or just after the decision of the fate of the infection process. Infected CV-1 cells were harvested and the nuclear lysates were subjected to ChIP with antibodies against p53, Sp1, T-ag and a control IgG. The amounts of viral DNA precipitated by the respective antibodies were analyzed by quantitative PCR, using primers that span the GC-boxes and 2 of the 3 T-ag binding sites. SV40 DNA was used as an internal standard. The results, presented as log values in Figure 7C, demonstrate that p53 indeed bound to the viral DNA *in vivo*. Notably p53-DNA binding is at approximately the same level at 10 and 16 hours post infection, precipitating 38.8 and 34.7 pg DNA per 2x10^5^ cells respectively (*p*-value = 0.4). On the other hand the binding of Sp1 increased ~2 fold, during that time period, from 8.3 to 16.9 pg (*p*-value < 0.05). Binding of T-ag to the promoter region increased over 21 fold between 10 and 16 hours post infection, precipitating 8.7 pg and 184.6 pg DNA respectively. This rise is consistent with the high rate of SV40 DNA replication during those 6 hours, which was ~20 fold (from 0.3 to 6 ng per 2x10^5^ cells). Taken together our results suggest that elevation of p53 in the first hours post infection leads to suppression of T-ag transcription through its binding to the early SV40 promoter, blocking progression of the infection.

## DISCUSSION

Since its discovery 35 years ago, p53 has emerged as a key player in cellular stress responses. Mostly known for its tumor suppressor role, it has also been implicated in protecting cells from a wide range of insults, including viral infection [6, 10, 61].

The present study extends the large list of viruses antagonized by p53 to include SV40. The experiments were conducted in two representative cell lines, human and monkey. While the monkey is the native host for SV40, and monkey derived cell-lines are fully permissive for SV40 infection, human cells are semi-permissive, supporting viral propagation at a lower efficiency [75]. Our recent experiments in the human MCF7 cells showed that the virus expresses T-ag and produces progeny, at a somewhat lower efficiency compared to monkey cells [59].

p53 was reported to be activated by viral replication, which induces DNA damage response [76, 77], or by specific viral proteins that are expressed following the infection [78]. SV40, however, elicits p53 host defense by a yet unknown mechanism.

We demonstrated that p53 activation confers protection against SV40 infection. In CV-1 cells, increasing p53 level through chemical inhibition of Mdm2 caused a significant decrease in viral infection. Consistent with that finding, shRNA-mediated knockdown of p53 in MCF7 cells led to a dramatic increase in the percentage of productively infected cells. Finally, the single-cell experiments unambiguously demonstrated that elevation of p53 at the beginning of the infection completely blocked T-ag expression and progress of the infection. Note that these studies suggest the existence of additional cell protection factors, since SV40 infection was also arrested in many of the p53 negative cells. Another possibility is that the endogenous p53 accounts for at least some of the host protective effect in cells that do not show a p53-venus signal upon infection. It is worth pointing out that at the multiplicity of infection used in the present study (30) the virus enters most of the infected MCF7 cells.

The study suggests that p53 defends the host by preventing T-ag protein expression (Figures 2 and 3). p53 was found to repress transcription from the early SV40 promoter by disrupting Sp1 binding in a dose-dependent manner (Figure 4 a,b in [72]). The DNA element interspersed within the six GC-boxes, which bind both Sp1 and p53, was identified as a pattern of helical twist angles, termed pyrimidine sandwich elements (PSEs), present in promoters of many viruses [79]. This element is associated with many cellular and viral promoters. Whether the downregulation by p53 is due to competition between the two regulators in DNA binding or to hetero-complex formation between Sp1 and p53 is not clear.

**Figure 8:**
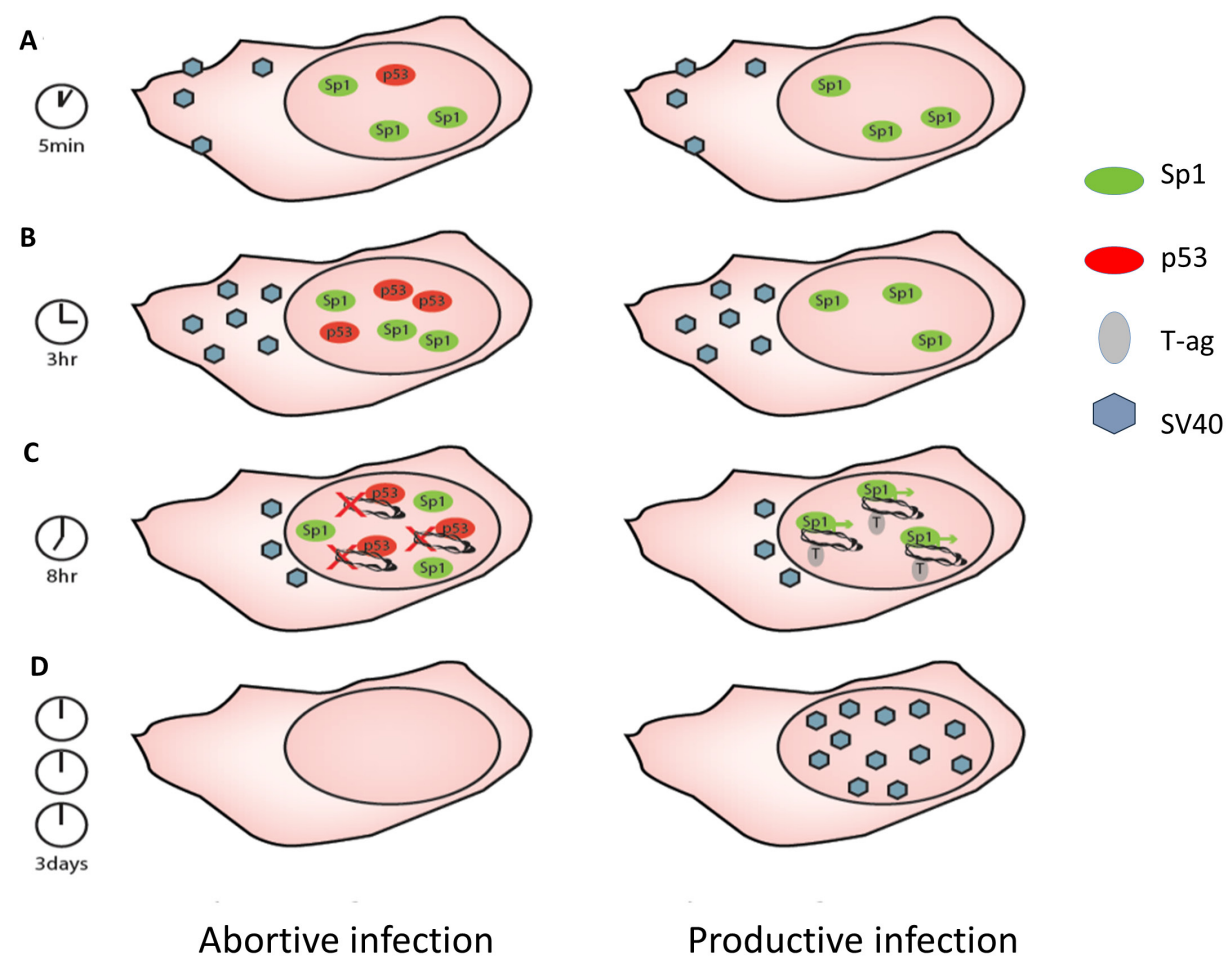
Model for the mechanism of p53 host defense against SV40 infection **A.** Cells infected by SV40 have variable levels of p53, due to sporadic spontaneous pulses of the protein as was previously reported. Cells with higher levels of p53 are primed toward its activation after the infection (cell on the left). Sp1 is presumed to be present in all cells. **B.** Activation of p53 start around 3 hours post infection, preferably in cells in which the protein is already above steady state level. **C.** Nuclear entry at 8-9 hours. The active p53 (left cell) captures the DNA and inhibits SV40 gene expression. In the absence of active p53 (right cell) T-ag is expressed and the viral DNA replicates. **D.** Virion particles are produced only in the right cell. Our model proposes that the decision between abortive vs productive infection occurs upon nuclear entry of the viral DNA.

We have previously demonstrated that individual proliferating cells exhibit spontaneous p53 pulses above its background expression level [23], as also seen in the present data of the mock-infected cells. We propose a model (see Figure 8) in which p53 is preferentially activated by the infecting virus in those cells that are already primed for its expression (as shown in Figure 8). Based on the ChIP and T-ag mRNA data (Figure 7), we propose that in cells where p53 is activated, p53 binds to the viral GC-boxes, which overlap the Sp1 binding sites, thereby repressing Tag expression. Importantly, *in vitro* studies demonstrated that p53 competed with Sp1 on binding to the SV40 early promoter, as well as many other promoters [69, 70]. We found that *in vivo* p53 binds to SV40 DNA during or soon after viral DNA entry into the nucleus (Figure 7). We propose that repression of T-ag expression at that time blocks progression of the infection in those cells (Figure 8). At the same time *in vivo* binding of Sp1 increases, suggesting that in cells with low p53 Sp1 binds to the newly replicated DNA, thereby increasing the T-ag transcription and the infection cycle. We propose that competition between p53 and Sp1 binding at the early SV40 promoter, as demonstrated earlier [69, 70], is at the core of the p53 protection mechanism.

Our results of the single cell studies supplement a growing body of work, showing that information processing by individual cells within a population may differ due to characterized and uncharacterized molecular events, as well as stochastic effects, leading to a wide variability in cell-to cell phenotype [80-82]. Hence the virus infects a heterogeneous cell population with respect to p53 level and presumable additional unknown cellular variables, which profoundly affect the outcome of infection.

In spite of p53 activation, mRNA of its target genes were not induced at the critical time window, 6-12 hours post infection, consistent with the lack of phosphorylation of S15, at the p53 transactivation domain. On the other hand S392 phosphorylation rises sharply at 8-9 hours. The rise follows the time of nuclear entry of the viral DNA at 7-8 hours, as defined by our previous studies [44] and supported by the present data: T-ag mRNA is still not visible at 7 hrs post infection and the protein starts appearing at 9 hours. These findings led us to propose that the decision between abortive and productive infection occurs as soon as the genome enters the nucleus. S392 phosphorylation enhances the DNA-binding affinity of p53, but not its transcriptional activity. In this context it is interesting that wild-type p53 has been reported to also inhibit *in vitro* T-ag dependent SV40 DNA replication by blocking the unwinding activity of T-ag [83]. Therefore it is possible that the infection in cells with excess p53 may be blocked even after T-ag began expression.

In the productively infected cells T-ag manipulates the host DNA replication machinery, reprogramming the cell for viral DNA replication [84]. T-ag was demonstrated to drive infected cells into aberrant DNA replication [85] by leading to activation of superfluous replication origins [86]. In addition, it is phosphorylated by the DNA damage signaling kinase ATM (ataxia telangiectasia-mutated) [87]. Notably, studies of cell populations demonstrated that ectopically expressed T-ag led to stabilization and accumulation of p53 [88]. Consistent with those studies, our single cell experiments show that late in the infection, when T-ag is present in substantial amounts, p53 accumulates in a significant proportion of the productively infected cells, albeit not in all. In spite of its accumulation, the function of p53 is blocked by T-ag via multiple mechanisms: by p53 sequestration, by blocking the site-specific DNA binding of p53 [60], or, in the absence of protein-protein complex formation, via activity of the J-domain of T-ag residing at its amino terminus [89]. Thus the multifaceted struggle between SV40 large T-ag and p53 that begins as soon as the viral DNA enters the nucleus via repression of T-ag transcription by p53, continues with the inactivation of p53 by T-ag through the progress of the viral life cycle.

We propose that during infection, although SV40 does not propagate in all cells, it produces sufficiently high progeny virions that are capable of infecting the surrounding, previously p53-protected cells. This may account for the complete lysis of SV40-infected cells observed in tissue culture experiments. Thus, while the mechanism underlying cellular heterogeneity is not understood, it appears that under laboratory conditions the virus has the upper hand in its battle against the host. In nature, however, host immune response as well as other factors may influence the outcome of the infection, which is manifested in the native monkey host as mild viremia or persistent latent infection.

An increasing number of the human polyomaviruses are being identified as human pathogens. The viruses are mostly widespread in the human population but are activated in immune-suppressed individual. BK may be activated following kidney or bone marrow transplantation leading to rejection as well as other complications [90]. Likewise, patients with underlying diseases and therapies that modulate the immune system are at high risk for activation of JC, leading to demyelinating disease, progressive multifocal leukoencephalopathy, PML [91]. Recently, personalized T-cell therapy targeted against the virus has been suggested as a promising treatment for these diseases [92]. An intriguing question is whether a similar p53-dependent host defense strategy operates in infections by pathogenic members of polyomaviridae, as well as other pathogens such as Herpes simplex, Cytomegalovirus and HIV [70]. Such findings will suggest novel clinical approaches that will be based on upregulation and/or activation of p53, for example by low dose irradiation.

Interestingly, the complex interaction between p53 and Sp1 in decision of the outcome of SV40 infection represents a simplified molecular model of cancer development. Sp1 is overexpressed in many types of cancer, including those carrying wild-type p53, and is considered, among other proteins, as a ‘hallmark of cancer’ [93]. Consequently, Sp1 over expression is associated with poor prognosis and it is therefore an attractive target for anticancer therapy [94]. External conditions that lead to tumorogenesis in p53 positive cancers may be investigated using the experimental system described here.

## MATERIALS AND METHODS

### Cell culture

CV-1 (ATCC #CCL70) are African Green monkey kidney cells. MCF-7 (ATCC # HTB-22) are human breast cancer cells. The p53^+^ and p53^-^ MCF-7 cells were previously described [57]. Cells were cultured in high glucose Dulbecco's modified Eagle's medium containing glutamine, penicillin, streptomycin and 10% FCS. MCF-7 cells carrying the p53-venus fusion protein were cultured in RPMI+10% FCS, with penicillin, streptomycin and G418 and were previously described [23, 25].

### SV40 production, purification and infection

SV40 was prepared by the di-detergent method as previously described [39]. Virus titer was measured by infecting CV-1 cells with serial dilutions and monitoring by FACS the percent of T-ag expressing cells [56]. Infections were performed when the monolayers were confluent, as described [39]; CV-1 cells were infected at moi 10 and MCF7 cells were infected at moi 30. To synchronize the infection, virus was allowed to adsorb for 30 min at 4°C. Time 0 designates the end of the adsorption period, when cells are washed from non-adsorbed virus, medium containing 10% FCS is added and the cultures are transferred to 37°.

### Antibodies and reagents

Primary antibodies for western blotting and for immune-histochemistry staining were anti-p53 DO-1 mouse monoclonal, (SC-126, Santa Cruz Biotechnology), Anti-p53 Pi-S392 rabbit monoclonal (ab134190), p53 Pi-S15 (ab1431) from ABCAM. Mouse monoclonal BH3 used for VP1 staining [95] was obtained from Ed Goodwin.

Secondary antibodies for western blotting were anti-Rabbit-HRP conjugated (ABCAM, ab97051), anti mouse-HRP conjugated (K4001, DAKO Agilent Technologies). Anti-GAPDH HRP-conjugated rabbit polyclonal (ab9385 ABCAM) was used for loading control. Secondary antibodies for immune-histochemistry were from Jackson ImmunoResearch Laboratories: Donkey anti-rabbit DyLight 488 (711485152) and donkey anti-mouse DyLight 649 (715495150). ChiP experiments were conducted with anti-p53 mouse monoclonal pAB1801 (ab28), anti-Sp1 (ChIP grade, ab13370), anti-Tag (ab416) and mouse IgG1, kappa monoclonal (MOPC-21, ab18443) for isotype control, all from ABCAM. Antibodies were used at concentrations recommended by the suppliers, following fine calibration.

Nutlin3 was purchased from Sigma-Aldrich (Cat No. N6287).

### Proteomics screen

“Signal Transduction Antibody Arrays” (Hypromatrix) were used based on the manufacturer's protocol (http://www.hypromatrix.com) with some modifications as previously described [39].

### Protein analyses

Whole cell lysates were prepared using RIPA buffer [96]. After removal of cell debris by centrifugation, 20 μg of protein were loaded on each lane of NuPAGE 4-12% Bis-Tris-Gel (Invitrogen). Polyacrylamide gel electrophoresis (PAGE) was followed by Western blot analyses with respective antibodies. The bands were quantified using ImageJ software (developed at the NIH). The images of the Western blots in Figure 2A were adjusted by Adobe Photoshop CS6. For the top panels (p53) brightness was increased by 150 and contrast by 100, and for the bottom panels (GAPDH) by 70 and 25.486 The GAPDH image In Figure 2C was also adjusted by increasing brightness by 150 and contrast by 100.

### Immunostaining

Cells grown and infected on cover slips were fixed and permeabilize d with 100% cold methanol for 20 minutes at -20ºC. The cells were washed 3 times with cold PBS, incubated with primary antibodies for 1 hr at RT, washed 3 times with Tween-PBS and once more with PBS, and stained with fluorophore-conjugated secondary antibodies for 30 min at 37°C. The primary antibodies were applied at 1:100 dilution and the secondary antibodies at 1:400. Images were acquired with a Zeiss LSM710 confocal microscope, using X40 lens.

### Measurement of T-ag positive cells by flow cytometry

Flow cytometry was done as previously described [56]. Infected cells were collected by trypsinization 48 hours post infection. T-ag was stained with AlexaFluor488-conjugated anti-mouse. Fluorescence of 10,000 cells was detected using the FL-1 sensor of a BD FACSCalibur, and the data analyzed by Cyflogic software.

### Time-lapse microscopy

MCF7 cells expressing p53-venus fusion protein were seeded on MatTek 6-well plates in transparent RPMI medium with 5% FCS. Cells were mock or SV40-infected, washed and placed in a climate control chamber inside the microscope. Images were acquired at 20 minutes intervals for 48 hours. The data obtained from time-lapse movies was analyzed using Matlab scripts developed in house.

### ChIP experiments

ChIP was performed as described by Carey et al [97], except that the cells were harvested by trypsinization. Cross-linking was performed by adding 1% formaldehyde in PBS to the cell pellet. Aliquots of 100 μl, corresponding to ~2x10^5^ cells, and 3 μg antibody were used for each ChIP assay.

### Quantitative real-time PCR of mRNA and viral DNA

For mRNA analysis, cells were collected by trypsinization and RNA extracted using the total RNA mini-kit (Geneaid) and DNase treatment. 2 μg total RNA was reverse-transcribed into cDNA using the High Capacity cDNA Reverse Transcription Kit (Applied Biosystems). The cDNA was mixed with SYBR Green master mix (Applied Biosystems) and with primers, and dispensed in triplicates onto a 384-well or 96-well plate, with HPRT RNA as internal standard.

PCR was performed on a QuantStudio or Step-One-Plus real-time PCR machines.

The forward primer for measuring the level of T-ag mRNA was

SV40 DNA extracted from the ChIP precipitates was measured similarly. The primers for the PCR reaction, CCAGGCACTCCTTTCAAGACC GCAACCAGGTGTGGAAAGTCC, span the *ori* SV40

## SUPPLEMENTARY MATERIALS FIGURES AND TABLES



## References

[1] Lane DP, Crawford LV (1979). T antigen is bound to a host protein in SV40-transformed cells. Nature.

[2] Linzer DI, Levine AJ (1979). Characterization of a 54K dalton cellular SV40 tumor antigen present in SV40-transformed cells and uninfected embryonal carcinoma cells. Cell.

[3] Levine AJ (1989). The p53 tumor suppressor gene and gene product. Princess Takamatsu symposia.

[4] Stanbridge EJ (1989). The evidence for human tumor suppressor genes. Princess Takamatsu symposia.

[5] Lane DP (1992). Cancer. p53, guardian of the genome. Nature.

[6] Levine AJ (1997). p53, the cellular gatekeeper for growth and division. Cell.

[7] Lane D, Levine A (2010). p53 Research: the past thirty years and the next thirty years. Cold Spring Harbor perspectives in biology.

[8] Farmer G, Bargonetti J, Zhu H, Friedman P, Prywes R, Prives C (1992). Wild-type p53 activates transcription *in vitro*. Nature.

[9] Toledo F, Wahl GM (2006). Regulating the p53 pathway: *in vitro* hypotheses, *in vivo* veritas. Nature reviews Cancer.

[10] Levine AJ, Oren M (2009). The first 30 years of p53: growing ever more complex. Nature reviews Cancer.

[11] Kracikova M, Akiri G, George A, Sachidanandam R, Aaronson SA (2013). A threshold mechanism mediates p53 cell fate decision between growth arrest and apoptosis. Cell death and differentiation.

[12] Das S, Boswell SA, Aaronson SA, Lee SW (2008). P53 promoter selection: choosing between life and death. Cell cycle (Georgetown, Tex).

[13] Kruse JP, Gu W (2008). SnapShot: p53 posttranslational modifications. Cell.

[14] Tang Y, Luo J, Zhang W, Gu W (2006). Tip60-dependent acetylation of p53 modulates the decision between cell-cycle arrest and apoptosis. Mol Cell.

[15] Tang Y, Zhao W, Chen Y, Zhao Y, Gu W (2008). Acetylation is indispensable for p53 activation. Cell.

[16] Batchelor E, Loewer A, Mock C, Lahav G (2011). Stimulus-dependent dynamics of p53 in single cells. Mol Syst Biol.

[17] Purvis JE, Karhohs KW, Mock C, Batchelor E, Loewer A, Lahav G (2012). p53 dynamics control cell fate. Science (New York, NY).

[18] Lavin MF, Gueven N (2006). The complexity of p53 stabilization and activation. Cell death and differentiation.

[19] Haupt Y, Maya R, Kazaz A, Oren M (1997). Mdm2 promotes the rapid degradation of p53. Nature.

[20] Kubbutat MH, Jones SN, Vousden KH (1997). Regulation of p53 stability by Mdm2. Nature.

[21] Prives C (1998). Signaling to p53: breaking the MDM2-p53 circuit. Cell.

[22] Michael D, Oren M (2003). The p53-Mdm2 module and the ubiquitin system. Seminars in cancer biology.

[23] Loewer A, Batchelor E, Gaglia G, Lahav G (2010). Basal dynamics of p53 reveal transcriptionally attenuated pulses in cycling cells. Cell.

[24] Lahav G, Rosenfeld N, Sigal A, Geva-Zatorsky N, Levine AJ, Elowitz MB, Alon U (2004). Dynamics of the p53-Mdm2 feedback loop in individual cells. Nat Genet.

[25] Batchelor E, Mock CS, Bhan I, Loewer A, Lahav G (2008). Recurrent initiation: a mechanism for triggering p53 pulses in response to DNA damage. Mol Cell.

[26] Reed SI, Stark GR, Alwine JC (1976). Autoregulation of simian virus 40 gene A by T antigen. Proc Natl Acad Sci U S A.

[27] Keller JM, Alwine JC (1984). Activation of the SV40 late promoter: direct effects of T antigen in the absence of viral DNA replication. Cell.

[28] Gordon-Shaag A, Yoseph Y, Abd-el-Latif M, Oppenheim A (2003). The abundant nuclear enzyme PARP participates in the life cycle of SV40 and is stimulated by the virus minor capsid protein VP2/3. J Virol.

[29] Raghava S, Giorda KM, Romano FB, Heuck AP, Hebert DN (2011). The SV40 late protein VP4 is a viroporin that forms pores to disrupt membranes for viral release. PLoS Pathog.

[30] Pinto M, Dobson S (2014). BK and JC virus: a review. The Journal of infection.

[31] White MK, Gordon J, Khalili K (2013). The rapidly expanding family of human polyomaviruses: recent developments in understanding their life cycle and role in human pathology. PLoS Pathog.

[32] Samimi M, Gardair C, Nicol JT, Arnold F, Touze A, Coursaget P (2015). Merkel cell polyomavirus in merkel cell carcinoma: clinical and therapeutic perspectives. Seminars in oncology.

[33] van der Meijden E, Janssens RW, Lauber C, Bouwes Bavinck JN, Gorbalenya AE, Feltkamp MC (2010). Discovery of a new human polyomavirus associated with trichodysplasia spinulosa in an immunocompromized patient. PLoS Pathog.

[34] Tsai B, Gilbert JM, Stehle T, Lencer W, Benjamin TL, Rapoport TA (2003). Gangliosides are receptors for murine polyoma virus and SV40. Embo J.

[35] Campanero-Rhodes MA, Smith A, Chai W, Sonnino S, Mauri L, Childs RA, Zhang Y, Ewers H, Helenius A, Imberty A, Feizi T (2007). N-glycolyl GM1 ganglioside as a receptor for simian virus 40. J Virol.

[36] Neu U, Woellner K, Gauglitz G, Stehle T (2008). Structural basis of GM1 ganglioside recognition by simian virus 40. Proc Natl Acad Sci U S A.

[37] Drayman N, Glick Y, Ben-nun-shaul O, Zer H, Zlotnick A, Gerber D, Schueler-Furman O, Oppenheim A (2013). Pathogens use structural mimicry of native host ligands as a mechanism for host receptor engagement. Cell host & microbe.

[38] Pelkmans L, Fava E, Grabner H, Hannus M, Habermann B, Krausz E, Zerial M (2005). Genome-wide analysis of human kinases in clathrin- and caveolae/raft-mediated endocytosis. Nature.

[39] Butin-Israeli V, Drayman N, Oppenheim A (2010). Simian virus 40 infection triggers a balanced network that includes apoptotic, survival, and stress pathways. J Virol.

[40] Engel S, Heger T, Mancini R, Herzog F, Kartenbeck J, Hayer A, Helenius A (2011). Role of endosomes in simian virus 40 entry and infection. J Virol.

[41] Norkin LC, Anderson HA, Wolfrom SA, Oppenheim A (2002). Caveolar endocytosis of simian virus 40 is followed by brefeldin A-sensitive transport to the endoplasmic reticulum, where the virus disassembles. J Virol.

[42] Schelhaas M, Malmstrom J, Pelkmans L, Haugstetter J, Ellgaard L, Grunewald K, Helenius A (2007). Simian Virus 40 depends on ER protein folding and quality control factors for entry into host cells. Cell.

[43] Daniels R, Rusan NM, Wadsworth P, Hebert DN (2006). SV40 VP2 and VP3 insertion into ER membranes is controlled by the capsid protein VP1: implications for DNA translocation out of the ER. Mol Cell.

[44] Butin-Israeli V, Ben-Nun-Shaul O, Kopatz I, Adam SA, Shimi T, Goldman RD, Oppenheim A (2011). Simian virus 40 induces lamin A/C fluctuations and nuclear envelope deformation during cell entry. Nucleus.

[45] Kobiler O, Drayman N, Butin-Israeli V, Oppenheim A (2012). Virus strategies for passing the nuclear envelope barrier. Nucleus.

[46] Hupp TR, Meek DW, Midgley CA, Lane DP (1992). Regulation of the specific DNA binding function of p53. Cell.

[47] Ullrich SJ, Sakaguchi K, Lees-Miller SP, Fiscella M, Mercer WE, Anderson CW, Appella E (1993). Phosphorylation at Ser-15 and Ser-392 in mutant p53 molecules from human tumors is altered compared to wild-type p53. Proc Natl Acad Sci U S A.

[48] Kapoor M, Lozano G (1998). Functional activation of p53 via phosphorylation following DNA damage by UV but not gamma radiation. Proc Natl Acad Sci U S A.

[49] Sakaguchi K, Sakamoto H, Lewis MS, Anderson CW, Erickson JW, Appella E, Xie D (1997). Phosphorylation of serine 392 stabilizes the tetramer formation of tumor suppressor protein p53. Biochemistry.

[50] Unger T, Sionov RV, Moallem E, Yee CL, Howley PM, Oren M, Haupt Y (1999). Mutations in serines 15 and 20 of human p53 impair its apoptotic activity. Oncogene.

[51] Walton MI, Wilson SC, Hardcastle IR, Mirza AR, Workman P (2005). An evaluation of the ability of pifithrin-alpha and -beta to inhibit p53 function in two wild-type p53 human tumor cell lines. Mol Cancer Ther.

[52] Sohn D, Graupner V, Neise D, Essmann F, Schulze-Osthoff K, JÃ¤nicke RU (2009). Pifithrin-alpha protects against DNA damage-induced apoptosis downstream of mitochondria independent of p53. Cell death and differentiation.

[53] Vassilev LT, Vu BT, Graves B, Carvajal D, Podlaski F, Filipovic Z, Kong N, Kammlott U, Lukacs C, Klein C, Fotouhi N, Liu EA (2004). *In vivo* activation of the p53 pathway by small-molecule antagonists of MDM2. Science (New York, NY).

[54] Sturm D, Montenarh M (1994). Expression of p53 after sv40 virus-infection of quiescent cells. Int J Oncol.

[55] Tiemann F, Deppert W (1994). Stabilization of the tumor suppressor p53 during cellular transformation by simian virus 40: influence of viral and cellular factors and biological consequences. J Virol.

[56] Drayman N, Kler S, Ben-nun-Shaul O, Oppenheim A (2010). Rapid method for SV40 titration. J Virol Methods.

[57] Brummelkamp TR, Bernards R, Agami R (2002). A system for stable expression of short interfering RNAs in mammalian cells. Science (New York, NY).

[58] Black PH, Crawford EM, Crawford LV (1964). The Purification of Simian Virus 40. Virology.

[59] Bauman Y, Drayman N, Ben-Nun-Shaul O, Vitenstein A, Yamin R, Ophir Y, Oppenheim A, Mandelboim O (2016). Downregulation of the stress-induced ligand ULBP1 following SV40 infection confers viral evasion from NK cell cytotoxicity. Oncotarget.

[60] Bargonetti J, Reynisdottir I, Friedman PN, Prives C (1992). Site-specific binding of wild-type p53 to cellular DNA is inhibited by SV40 T antigen and mutant p53. Genes & development.

[61] Rivas C, Aaronson SA, Munoz-Fontela C (2010). Dual Role of p53 in Innate Antiviral Immunity. Viruses.

[62] Martin RG, Chou JY, Avila J, Saral R (1975). The semiautonomous replicon: a molecular model for the oncogenicity of SV40. Cold Spring Harbor symposia on quantitative biology.

[63] Munoz-Fontela C, Macip S, Martinez-Sobrido L, Brown L, Ashour J, Garcia-Sastre A, Lee SW, Aaronson SA (2008). Transcriptional role of p53 in interferon-mediated antiviral immunity. J Exp Med.

[64] Rathi AV, Cantalupo PG, Sarkar SN, Pipas JM (2010). Induction of interferon-stimulated genes by Simian virus 40 T antigens. Virology.

[65] Giacobbi NS, Gupta T, Coxon AT, Pipas JM (2015). Polyomavirus T antigens activate an antiviral state. Virology.

[66] Dharel N, Kato N, Muroyama R, Taniguchi H, Otsuka M, Wang Y, Jazag A, Shao RX, Chang JH, Adler MK, Kawabe T, Omata M (2008). Potential contribution of tumor suppressor p53 in the host defense against hepatitis C virus. Hepatology (Baltimore, Md).

[67] Shen Y, Wang X, Guo L, Qiu Y, Li X, Yu H, Xiang H, Tong G, Ma Z (2009). Influenza A virus induces p53 accumulation in a biphasic pattern. Biochemical and biophysical research communications.

[68] Laub O, Aloni Y (1975). Transcription of simian virus 40. V. Regulation of simian virus 40 gene expression. J Virol.

[69] Jackson P, Bos E, Braithwaite AW (1993). Wild-type mouse p53 down-regulates transcription from different virus enhancer/promoters. Oncogene.

[70] Bargonetti J, Chicas A, White D, Prives C (1997). p53 represses Sp1 DNA binding and HIV-LTR directed transcription. Cellular and molecular biology (Noisy-le-Grand, France).

[71] Dynan WS, Tjian R (1983). The promoter-specific transcription factor Sp1 binds to upstream sequences in the SV40 early promoter. Cell.

[72] Bargonetti J, Friedman PN, Kern SE, Vogelstein B, Prives C (1991). Wild-type but not mutant p53 immunopurified proteins bind to sequences adjacent to the SV40 origin of replication. Cell.

[73] Perrem K, Rayner J, Voss T, Sturzbecher H, Jackson P, Braithwaite A (1995). p53 represses SV40 transcription by preventing formation of transcription complexes. Oncogene.

[74] Chou JY, Avila J, Martin RG (1974). Viral DNA synthesis in cells infected by temperature-sensitive mutants of simian virus 40. J Virol.

[75] Small MB, Gluzman Y, Ozer HL (1982). Enhanced transformation of human fibroblasts by origin-defective simian virus 40. Nature.

[76] Weitzman MD, Carson CT, Schwartz RA, Lilley CE (2004). Interactions of viruses with the cellular DNA repair machinery. DNA repair.

[77] Lilley CE, Schwartz RA, Weitzman MD (2007). Using or abusing: viruses and the cellular DNA damage response. Trends in microbiology.

[78] Sato Y, Tsurumi T (2013). Genome guardian p53 and viral infections. Reviews in medical virology.

[79] MacLeod MC (1993). Identification of a DNA structural motif that includes the binding sites for Sp1, p53 and GA-binding protein. Nucleic acids research.

[80] Altschuler SJ, Wu LF (2010). Cellular heterogeneity: do differences make a difference?. Cell.

[81] Loewer A, Lahav G (2011). We are all individuals: causes and consequences of non-genetic heterogeneity in mammalian cells. Curr Opin Genet Dev.

[82] Snijder B, Pelkmans L (2011). Origins of regulated cell-to-cell variability. Nat Rev Mol Cell Biol.

[83] Friedman PN, Kern SE, Vogelstein B, Prives C (1990). Wild-type, but not mutant, human p53 proteins inhibit the replication activities of simian virus 40 large tumor antigen. Proc Natl Acad Sci U S A.

[84] Zhao X, Madden-Fuentes RJ, Lou BX, Pipas JM, Gerhardt J, Rigell CJ, Fanning E (2008). Ataxia telangiectasia-mutated damage-signaling kinase- and proteasome-dependent destruction of Mre11-Rad50-Nbs1 subunits in Simian virus 40-infected primate cells. J Virol.

[85] Chou JY, Martin RG (1975). DNA infectivity and the induction of host DNA synthesis with temperature-sensitive mutants of simian virus 40. J Virol.

[86] Martin RG, Oppenheim A (1977). Initiation points for DNA replication in nontransformed and simian virus 40-transformed Chinese hamster lung cells. Cell.

[87] Shi Y, Dodson GE, Shaikh S, Rundell K, Tibbetts RS (2005). Ataxia-telangiectasia-mutated (ATM) is a T-antigen kinase that controls SV40 viral replication *in vivo*. J Biol Chem.

[88] Hein J, Boichuk S, Wu J, Cheng Y, Freire R, Jat PS, Roberts TM, Gjoerup OV (2009). Simian virus 40 large T antigen disrupts genome integrity and activates a DNA damage response via Bub1 binding. J Virol.

[89] Pipas JM, Levine AJ (2001). Role of T antigen interactions with p53 in tumorigenesis. Seminars in cancer biology.

[90] Pham PT, Schaenman J, Pham PC (2014). BK virus infection following kidney transplantation: an overview of risk factors, screening strategies, and therapeutic interventions. Current opinion in organ transplantation.

[91] Monaco MC, Major EO (2015). Immune System Involvement in the Pathogenesis of JC Virus Induced PML: What is Learned from Studies of Patients with Underlying Diseases and Therapies as Risk Factors. Frontiers in immunology.

[92] Mani J, Jin N, Schmitt M (2014). Cellular immunotherapy for patients with reactivation of JC and BK polyomaviruses after transplantation. Cytotherapy.

[93] Beishline K, Azizkhan-Clifford J (2015). Sp1 and the ‘hallmarks of cancer’. The FEBS journal.

[94] Vizcaino C, Mansilla S, Portugal J (2015). Sp1 transcription factor: A long-standing target in cancer chemotherapy. Pharmacology & therapeutics.

[95] Babe LM, Brew K, Matsuura SE, Scott WA (1989). Epitopes on the major capsid protein of simian virus 40. J Biol Chem.

[96] Ngoka LC (2008). Sample prep for proteomics of breast cancer: proteomics and gene ontology reveal dramatic differences in protein solubilization preferences of radioimmunoprecipitation assay and urea lysis buffers. Proteome science.

[97] Carey MF, Peterson CL, Smale ST (2009). Chromatin immunoprecipitation (ChIP). Cold Spring Harbor protocols.

