# Supplementary material for: p53 elevation in human cells halt SV40 infection by inhibiting T-ag expression
Source: Oncotarget. 2016 Jul 21;7(33):52643–60. doi: 10.18632/oncotarget.10769 (PMC5288138; doi:10.18632/oncotarget.10769)
Supplement: Supplementary file 1 [file oncotarget-07-52643-s001.pdf]

p53 elevation in human cells halt SV40 infection by inhibiting T-ag expression

Supplementary Material

Table S1. The effect of Nutlin on SV40 cell entry and productive infection

Treatment	Stained Protein	Nutlin	
		0	20 µM
SV40 infected	VP1	93.7 ± 1.3	83.3 ± 2.8
	Tag	78.5 ± 0.5	23.0 ± 1.0
Mock infected	VP1	0.4 ± 0.1	0.7 ± 0.1
	Tag	0.5 ± 0.0	1.4 ± 0.1

CV-1 cells were pre-treated with 20 µM Nutlin3 in 1% DMSO, or with 1% DMSO alone, for 16 hours before infection. The cells were infected with SV40 at moi=10 and the percentage of cells that were VP1 or T-ag positive was determined 3 hr or 24 hr post adsorption respectively, by antibody staining and FACS analysis.

Table S2. PCR primers for p53-target genes

	**Forward**	**Reverse**
p21	CTGGAGACTCTCAGGGTCGAA	GGCGTTTGGAGTGGTAGAAATCT
Bax	GCTGTTGGGCTGGATCCAAG	TCAGCATCTTCTTCCAGA
MxA	GCCGGCTGTGGATATGCTA	TTTATCGAAACATCTGTGAAAGCAA
OAS1	AGAAGGCAGCTCACGAAACC	CCACCACCCAAGTTTCCTGTA
IRF7	AAGAGCTGGTGGAATTCCGG	CAGGGAAGACACACCCTCAC
IRF9	GGATCAGAGGTCCCTGGAGT	TGAAGGTGAGCAGCAGTGAG
RIG-I	AGTGAGCATGCACGAATGAA	GGGATCCCTGGAAACACTTT

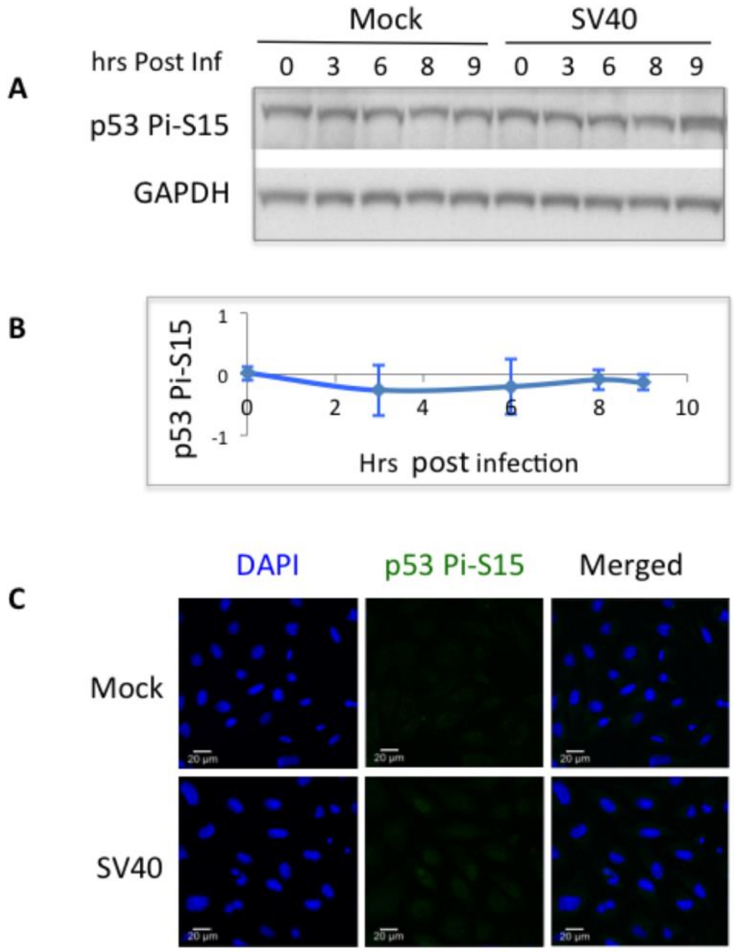

Figure S1. p53 S15 is not phosphorylated following SV40 infection. **A.** A representative western blot treated with antibody against p53 Pi-S15, showing that p53 is not phosphorylated following SV40 infection in comparison to the mock-infected cells. GAPDH is a loading control. **B.** Quantification of 3 blots demonstrating that there is no increase in phosphorylation of S15 following SV40 infection. The values of the quantified bands of 3 mock infections were subtracted from the corresponding bands of SV40 infection. The graph depicts average of 3 experiments; standard errors are represented by bars. **C.** Images of Mock and SV40-infected cells stained with DAPI, antibody against p53 Pi-S15 and merge.

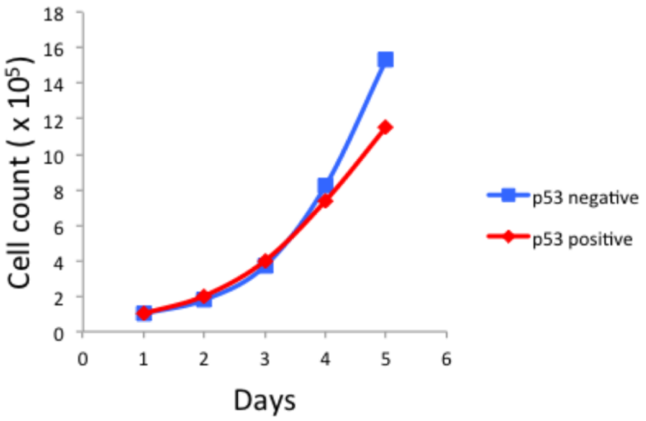

Figure S2. Growth rate of p53-positive and p-53 negative MCF7 cell lines. Each of the cell-lines was seeded in replicates at 10^5 cells per well in a 6-well plate. Live and dead cells counted every 24 hours for 5 additional days. Both cell-lines grew at the same rate for the first 4 days, when confluence of 70-75% was reached. Error bars are too small to be observed in the figure.

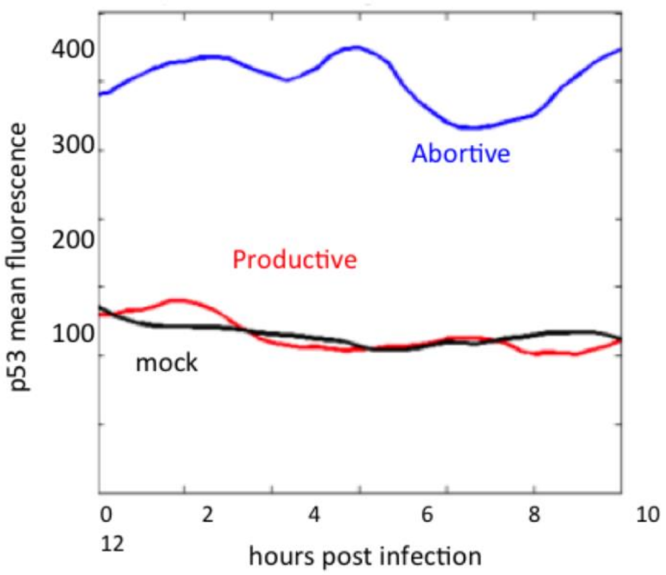

Figure S3. Dynamics of p53 in SV40-infected single cells. The data of average fluorescence of p53 of each group of cells, mock infected, abortively infected and productively infected, for the first 12 hours of the infection, shown in Fig. 3C, is presented here in an expanded form.

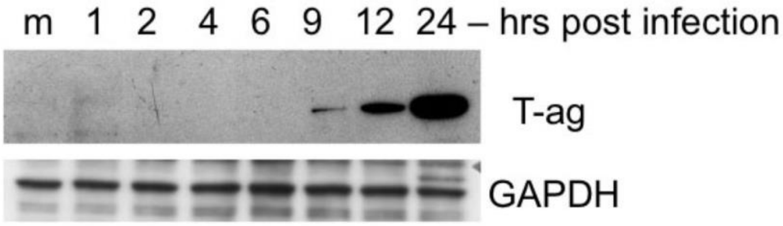

Figure S4. T-ag expression in CV-1 cells. Total protein extracts of infected CV-1 cells (moi 10) were analyzed for T-ag by western blotting. 2.5 µg protein were loaded on each lane.
